# Are the 50’s, the transition decade, in choroid plexus aging?

**DOI:** 10.1007/s11357-021-00329-x

**Published:** 2021-02-12

**Authors:** Ana Tahira, Fernanda Marques, Bianca Lisboa, Arthur Feltrin, André Barbosa, Kátia Cristina de Oliveira, Carlos Alberto de Bragança Pereira, Renata Leite, Lea Grinberg, Claudia Suemoto, Renata Eloah de Lucena Ferretti-Rebustini, Carlos Augusto Pasqualucci, Wilson Jacob-Filho, Helena Brentani, Joana Almeida Palha

**Affiliations:** 1 LIM23, Instituto de Psiquiatria, Hospital das Clínicas HCFMUSP, Faculdade de Medicina, Universidade de São Paulo, São Paulo, SP Brazil; 2 Life and Health Sciences Research Institute (ICVS), School of Medicine, University of Minho, Braga, Portugal; 3 ICVS/3B’s-PT Government Associate Laboratory, Braga/Guimarães, Portugal; 4 Center of Mathematics, Computing and Cognition, Federal University of ABC, Santo André, SP Brazil; 5 Inter-institutional Grad Program on Bioinformatics, University of São Paulo, São Paulo, SP Brazil; 6 Departamento de Estatistísca, Núcleo de Bioinformática, Instituto de Matemática, Universidade de São Paulo, São Paulo, SP Brazil; 7 Biobank for Aging Studies Group, Faculdade de Medicina, Universidade de São Paulo, São Paulo, SP Brazil; 8 Departamento de Psiquiatria, Faculdade de Medicina, Universidade de São Paulo, São Paulo, SP Brazil; 9 Clinical Academic Center, Braga, Portugal

**Keywords:** Choroid plexus, Human, Aging, High-throughput sequencing

## Abstract

The choroid plexus (CP) is an important structure for the brain. Besides its major role in the production of cerebrospinal fluid (CSF), it conveys signals originating from the brain, and from the circulatory system, shaping brain function in health and in pathology. Previous studies in rodents have revealed altered transcriptome both during aging and in various diseases of the central nervous system, including Alzheimer’s disease. In the present study, a high-throughput sequencing of the CP transcriptome was performed in postmortem samples of clinically healthy individuals aged 50’s through 80’s. The data shows an age-related profile, with the main changes occurring in the transition from the 50’s to the 60’s, stabilizing thereafter. Specifically, neuronal and membrane functions distinguish the transcriptome between the 50’s and the 60’s, while neuronal and axon development and extracellular structure organization differentiate the 50’s from the 70’s. These findings suggest that changes in the CP transcriptome occur early in the aging process. Future studies will unravel whether these relate with processes occurring in late- onset brain diseases.

**Supplementary Information:**

The online version contains supplementary material available at 10.1007/s11357-021-00329-x.

## Introduction

Increased life expectancy contributed to a marked increase in age-associated diseases. A better understanding on how organs normally age will contribute to bring additional healthy life years. In the brain, aging is a complex process that causes significant structural and physiological changes [1, 2]. Several studies have explored the impact of aging on brain physiology and behavior [2, 3], demonstrating age-related impairments on cognitive functions [4], exploratory and locomotor activities [5], sensorimotor behavior [6], and depressive-like states [7]. Accompanying these behavioral alterations in normal aging are structural changes of brain volume, cerebral blood flow, metabolic rate for oxygen consumption, glucose oxidation, blood volume, and cerebrospinal fluid (CSF) production [8]. Of interest, the CSF is mainly produced by the choroid plexus (CP), which is a thin membrane present within the brain ventricles and that is part of the brain barriers. The CP is formed by a monolayer of epithelial cells that surrounds and encloses a central stromal space rich in fenestrated blood vasculature (lacking the blood-brain barrier), thus not restricting the movement of molecules. However, diffusion into the CSF is prevented by the tight junctions that characterize the CP epithelial monolayer. Similarly to other brain regions, aged human CP also exhibits general cellular atrophy, decreased enzymatic and metabolic activities (and as such of CSF production), and impaired capacity for the efflux and clearance of molecules [9–12].

In the past few years, we and others have characterized, in rodents, the CP transcriptome in various ages and disease conditions. These studies showed upregulation of type I interferon (IFN-I)-dependent expression profile with aging [13], which was associated with the cognitive decline observed in older animals [14], and disturbance in the genes that modulate the cellular circadian cycle, transport of nutrients, and lipid metabolism [13]. These alterations support the idea that there is a clear dysfunction of the CP with aging, which impacts the production of CSF and the integrity of the blood-CSF barrier [13]. The present study intends to characterize the CP transcriptome in postmortem samples of clinically healthy humans from 50 to >80 years of age.

## Methods

### Participants

Samples were provided by the Biobank for Aging Studies from the Faculty of Medicine at the University of São Paulo (BBAS-USP), formerly known as the Brain Bank of the Brazilian Aging Brain Study Group (BBBABSG) [15], which is supplied by the São Paulo City Autopsy Service (SPAS). Study protocols were reviewed by the local ethics committee and approval granted by the FM-USP’s Institutional Review Board under protocol number 594/05, with all experiments performed in compliance with CAPPesq rules. All family members gave written informed consent for the use of the samples and clinical information.

### Neuropathological analysis

Neuropathological analysis was performed following the BBBABSG routine [15]. The brains were removed during autopsy within 4–20 h after death (postmortem interval in hours). One brain hemisphere was cut into 1-cm coronal slabs and sampled in 13 neurodegenerative disease-related structures, plus any other macroscopically lesioned areas, for microscopic examination. All sections were stained with hematoxylin and eosin. Immunohistochemistry was performed using antibodies against β-amyloid peptide (4G8; 1/5,000; Signet Laboratories, Dedhan, MA), phospho-tau (PHF-1; 1/ 1,000; gift of Peter Davies, New York, NY) and against α-synuclein (EQV-1; 1/10,000; gift of Kenji Ueda, Tokyo, Japan).

Neuropathological diagnoses were performed following internationally accepted guidelines. The Consortium to Establish a Register for Alzheimer’s disease criteria (CERAD) [16] were used to classify the β-amyloid neuritic plaque burden as none, scarce, moderate, or frequent. The distribution of neurofibrillary tangles (NFTs) was classified according to the Braak and Braak staging system [17] as stages I to VI. The usual neuropathological guidelines were used for Parkinson’s disease and other dementias [18].

The diagnosis of argyrophilic grain disease (AGD) was based on the presence of abundant phosphorylated tau-positive grains in the CA1 sector of the hippocampus, pretangles, especially in the hippocampal CA2 sector, and oligodendrocytes with coiled bodies in the hippocampal/temporal white matter [19]. The classification as healthy cases considered the absence or rare density of neuritic plaques (CERAD 0 or A) and a Braak stage up to III for neurofibrillary tangle distribution. Assessment of vascular lesion was performed by macroscopic examination.

In this study, the primary criterium to label a subject as healthy control was clinical (see below in functional assessments). It is of relevance to refer that subjects considered healthy have some level of neuropathological changes, expected to this age group, according to several independent postmortem cohorts. We excluded samples whenever they met neuropathological criteria for a neuropathological condition. It is commonly accepted that the diagnosis of Alzheimer’s disease (AD) requires AD neuropathology reaching scores A2B2C2 according to the most recent neuropathological guidelines [20]. Samples with any level of the Lewy body disease or significant vascular changes were not included. The cause of dead and information on chronic diseases and body mass index of each participant are provided in Table S1.

### Functional assessments

All individuals have a clinical dementia rate (CDR) equal to 0, showing no cognitive impairment [21]. The Informant Questionnaire on Cognitive Decline in the Elderly (IQCODE) [22, 23], which assesses functional cognitive status (in general the scores range from 3 to 5; for the Brazilian population, the cut off for dementia is of 3.42), was applied to family members [24].

### Statistical analysis

Statistical analysis of clinical and neuropathological variables was performed using likelihood function [25, 26]. Binomial probability was applied, and its value corresponded to samples size and frequency in the population. For those variables without previous information about population parameters of the variable, the median was used as a threshold to apply binomial distribution with probability of success equal to 0.5 [27]. For variables with more than two categories, the homogeneity test was performed [28].

### Sample preparation

After clinical and neuropathological selection, the CP material was obtained from the samples of the right lateral ventricle.

### RNA extraction and libraries construction

Total RNA extraction was performed using QIASymphony (Qiagen, CA, EUA) with miRNA CT 400 V8 QIAsymphony RNA kit (931636) QIAGEN (Qiagen) according to the manufacturer's instructions. RNA quality was measured using Bioanalyzer and all samples presented RNA Integrity Number (RIN) higher than 5. RNA-sequencing libraries were constructed using the TruSeq Stranded Total RNA Ribo-Zero Library Preparation Kit Illumina according to the manufacturer’s instructions and the paired-end sequencing was performed with the HiSeq 2500 (Illumina Inc., San Diego, CA, USA).

### RNA-sequencing analysis pipeline

Reads from RNA sequencing were quality checked with FASTQC (v0.11.2) [29] and adapters (Illumina PCR multiplex) and reads with low base quality were removed with FASTX (v0.0.13) [30]. Reads were aligned with HISAT2 (v2.1.0) [31, 32] using specific strand parameter and assembly GRCh38 from the Genome Reference Consortium (v84) as genome reference. GRCh38.84 Ensembl composed of 60,504 genes were used as reference for gene annotation in the alignment step. Qualimap2 (rnaseq v2.2.1) [33] and RSeQC (v2.6.4) [34] were used to check for quality mapping. The assembly of the transcripts was performed using StringTie (v1.3.3) [31] with the same gene reference annotation (GRCh38.84) previously used in the alignment. Filtering steps consisted in excluding transcripts that matched to rRNA and those that were not identified as being an existing transcript (=, transcripts that have same structure of annotation file) or new isoform (j, transcripts that harbor new structures of splicing junction, different from those in the annotation file). Gene expression was measured using HTSeq (v0.6.1) [35] with intersection-nonempty and strand specific parameters. Counts were converted to counts per million (CPM) using EdgeR (v3.20.7 - R 3.4.4) [36, 37] and only genes presenting a CPM ≥ 0.3 were considered for further analysis. To check for bias or outlier samples, Pearson’s correlation, hierarchical clustering with Euclidean distance, and principal component analysis (PCA) were performed. For gene expression comparison among groups, only genes that presented expression values in at least 50% of the samples in at least one of the groups were used. To search for hidden sources of variables, surrogate variable analysis (SVA, v3.26.0-R 3.4.4) [38] was used with normalized data by DESeq2 package (v1.18.1-R 3.4.4) [39]. In SVA analysis, age groups were considered as interest variable, and sex as adjustment variable. Negative binomial generalized linear models implemented in DESEq2 (v1.18.1 - R 3.4.4) were used for differential gene expression analysis using sex and surrogate variables (SVs) as covariables. The raw data of RNA-seq in this study is available in SRA repository under the number accession PRJNA515530 and study SRP179998.

### Co-expression analyses

Co-expression gene module analyses were performed using the weighted gene co-expression network analysis (WGCNA v0.1.63-R 3.3.1) [40, 41]. First, counting datasets were normalized with variance stabilizing transformation (VST) implemented in DESEq2 (v1.18.1-R 3.4.4) and 10% of the lowest expressed genes were excluded to reduce noise. Only genes that were expressed in at least 50% of samples in each group were considered for further analyses. Co-expression network analyses were applied to normalized expression data to build unsigned gene co-expression modules [42]. For instance, a Pearson’s correlation matrix was calculated to each gene pairwise, and an adjacency matrix was computed increasing the correlation matrix with appropriate power (β) which maximized the scale-free topology network (*R*^2^ fit above 0.8). Based on adjacency matrix, genes were grouped with average linkage hierarchical clustering according to topological overlap measures. Lastly, to define gene co-expressed modules, branches of the resulting tree were split using hybrid dynamic tree-cutting with a deepSplit parameter set to 2 and a minimum of 300 genes within the defined module. To compare gene modules changes among groups, a preservation analysis was performed using 100 permutations to account for the possibility of randomness in the comparison [43]. This analysis compared the topological measures of each gene module among groups and changes in module properties are reflected in *Z*-summary, which indicates whether a module was strongly (*Z*-summary ≥ 10), moderately (2≤*Z*-summary < 10) or not preserved (*Z*-summary < 2). The *Z*-summary and preservation median rank, which accounts for module size, were used as criteria to determine the least preserved modules in each analysis. Genes that presented k.ME ≥ 0.9 were defined as hub genes in each module.

## Results

### Sample characterization

Samples were collected from 15 individuals: 8 men (53%) and 7 women (47 %). All individuals in this study showed CDR = 0 and IQCODE < 3.08 and were independent in daily activities. Samples were clustered according to age, five samples in G50 (50–59 years), six samples in G60 (60–69 years) and four samples in G70 (≥70 years). Table 1 shows age, education, alcohol consumption, and clinical and neuropathological characteristics of the age groups. Taking into account that the probability of women in our population is 0.52, in G50, for example, there are two women in the universe of 5, the probability of this happening (Prob. *R*) in our population is 94%, meaning that this probability did not diverge from the expected proportion; as such, it is representative of that in the population, in the same way for G60 and G70. Similarly, all groups are identical with respect to alcohol and tobacco use and on clinical and neuropathological characteristics.

**Table 1:** Age, education, alcohol consumption, and clinical and neuropathological characteristics of the groups

Variable	Parameters	G50 (*n*=5)	G60 (*n*=6)	G70 (*n*=4)
Sex (%)	Female	2 (40)	2 (33)	3 (75)
	Male	3 (60)	4 (67)	1 (25)
	Prob. *R*	94.2	70.6	70.8
Education in years	Median	4	4	6
	Mean (SE)	4.8 (1.50)	4.17 (1.22)	5 (1.91)
	Prob. *R*	100	75	100
Alcohol abuse (%)	Never	2 (40)	3 (50)	2 (50)
	Other	3 (60)	3 (50)	2 (50)
	Prob. *R*	100	100	100
Tobacco abuse (%)	Never	0 (0)	3 (50)	2 (50)
	Other	5 (100)	3 (50)	2 (50)
	Prob. *R*	54.9	44.3	54.6
Postmortem interval in hours	Median	15:36	14:38	15:00
	Mean (SE)	14:33 (1:28)	14:58 (2:04)	12:18 (4:25)
	Prob. *R*	100	100	100
AGD (%)	Yes	1 (20)	1 (17)	3 (75)
	No	4 (80)	5 (83)	1 (25)
	Prob. *R*	100	100	0.12
CERAD (%)	0	4 (80)	5 (83)	3 (75)
	(A, B, C)	1 (20)	1 (17)	1 (25)
	Prob. *R*	100	100	100
NPI (%)	Median	5.05	8.35	3.79
	Mean (SE)	4 (2.26)	7.83 (3.41)	2.5 (1.89)
	Prob. *R*	100	100	66.7
Braak (%)*	I	1 (20)	0 (0)	0 (0)
	II	4 (80)	4 (67)	3 (75)
	III	0 (0)	2 (33)	1 (25)

*ssn* sample size; *n* frequency; *postmortem interval* the time interval between death and autopsy and consequent brain collection. Family members (or care taker informed) on education (in number of years), alcoholism (alcohol consumption (yes, no, and stopped) and frequency (social or alcoholism)) and smoking (use of tobacco (yes, no, or stopped))

*Homogeneity test was applied with LRT stat of 2.07 and *p* value of 0.72

### Differential CP expression

**Fig. 1:**
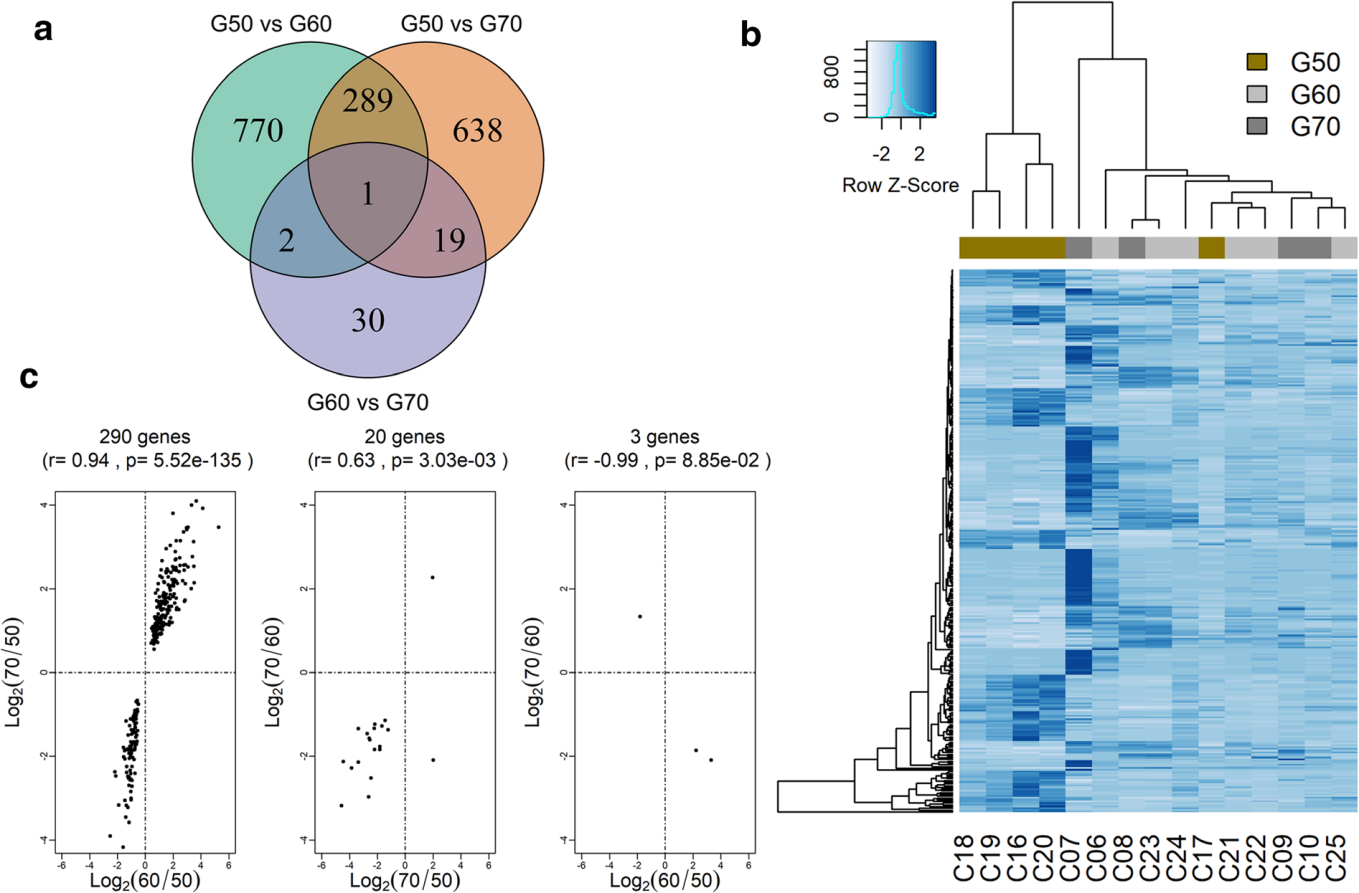
The Venn diagram of differentially expressed genes (DEGs) in each group comparison (**a**). Heatmap of 311 DEGs presented in at least two comparisons, rows represent genes and columns each sample. Bars are colored according to each group G50 (gold), G60 (light gray), and G70 (dark gray). Gene expressions are scaled by blue colors, and light blue indicates lower expression and dark blue higher expression (**b**). Plot of log2 (ratio expression) of overlapping gene between the comparison analysis (**c**)

Transcriptome sequencing from the 15 samples (G50 (*n*=5), G60 (*n*=6), G70 (*n*=4)) resulted in 380 million reads (25.4 million per sample) with an overall mapping rate to the human genome of 84%. Quality control regarding the gene coverage by read mapping showed no bias in distribution of reads along the gene (Figure S1). However, some samples showed large multiple mapping rates; thus, a stricter criterion to count genes was applied and those reads that had multiple mapping were disregarded. This approach decreased the number of genes engaged in the analyses but increased the correlation among samples (Figure S2). After applying this pipeline to filter out low-expressed genes (CPM ≥ 0.3), 15,319 genes were considered for further analyses. Expressed genes in each comparison, G50 vs G60 (*n* = 13,053 genes), G50 vs G70 (*n* = 12,674 genes), and G60 vs G70 (*n* = 14,890 genes), and differentially expressed genes were calculated. When comparisons were performed with a strict criterion (adjP ≤ 0.05), only those comparing with G50 resulted in genes differentially expressed. Therefore, a less stringent criteria (*p* value ≤ 0.01) was applied. This approach resulted in 1062 genes differentially expressed (DEGs) for G50 vs G60 (Table S2), being 669 up- and 393 downregulated in G60, 947 DEGs for G50 vs G70 (Table S2), being 483 up- and 464 downregulated in G70, and only 52 DEGs for G60 vs G70 (Table S2), being 15 up- and 37 downregulated in G70. Comparison among the three analyses showed only one overlapping gene (*TUBB4*) with higher expression in G60. Large overlapping was observed comparing DEGs from G50 vs G60 and G50 vs G70 (Fig. 1a), clustering analysis of DEGs showed high similarity in G50 samples which was not observed in G60 or G70 (Fig. 1b). All the common genes from this comparison presented concordant changes of expression, being up- or downregulated in G50 (Fig. 1c).

### Gene ontology enrichment analysis

Gene ontology and biological pathways analysis of differentially expressed genes, performed using the WebGestalt Software [44] (padj ≤ 0.05, minimum number of genes 5 and human genome as background), showed that the majority of the altered biological pathways are observed when comparing G50’s with G60’ or with G70’s, which suggests that most of the alterations occur in the transition of the 50’s into older ages. Supplementary data Table S3, S4 presents the complete list of altered pathways. Due to the low number of CP altered genes from G60 when compared with G70, only one biological pathway, extracellular structure organization (GO:0043062, FDR = 8.38E-03), encompassing 7 genes (COL1A1;COL1A2;LUM;SFRP2;THSD4;CRISPLD2;ADAMTS4), was found altered.

### Patterns of gene expression changings

**Fig. 2:**
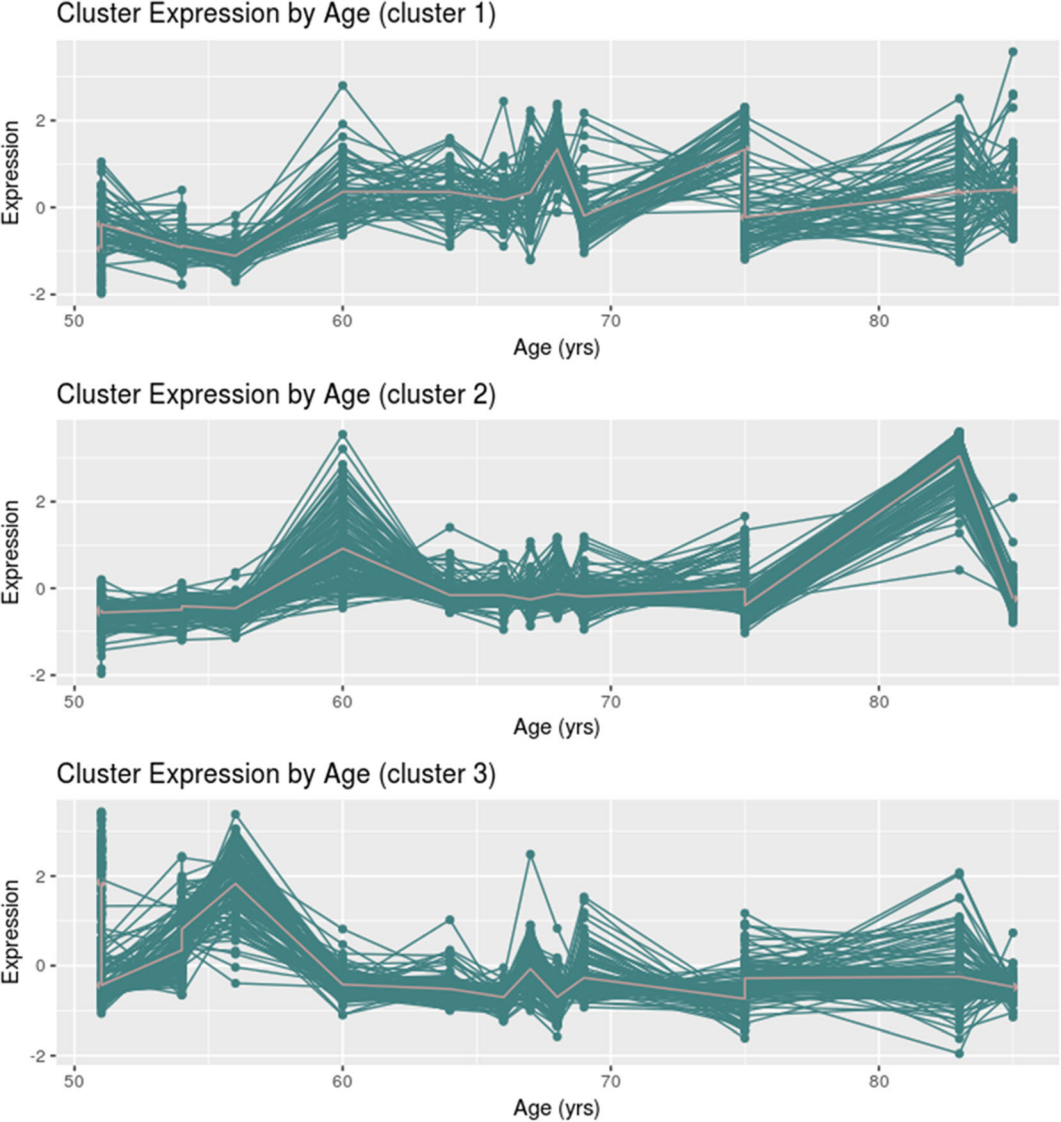
*k*-means clustering of genes differentially expressed in at least one comparison (*n* = 311 genes). The *x*-axis shows the sample ages, and *y*-axis represents the normalized expression counts value after *z*-score normalization. Cluster 1 (*n* = 60 genes), Cluster 2 (*n* = 134 genes), and Cluster 3 (*n*= 117 genes)

To verify the pattern of gene expression changing with age, we performed the *k*-means clustering using the DEGs among the age groups. The number of groups in *k*-means analysis was chosen according to the total within sum of squares (which shows the sum of variance of all groups); it resulted in *k*=3 (Figure S5). Therefore, *k*-means analysis showed three distinct patterns of expression in the group of DEGs (Figure 2). Cluster 1 is composed of 60 genes and their highest expression is represented in samples aged ranging 60 to 75 years. Cluster 2 is composed of 134 genes with highest expression represented in samples at 60 years and after 75 years and Cluster 3 with 117 genes and highest expression during 50 to 60 years.

**Fig. 3:**
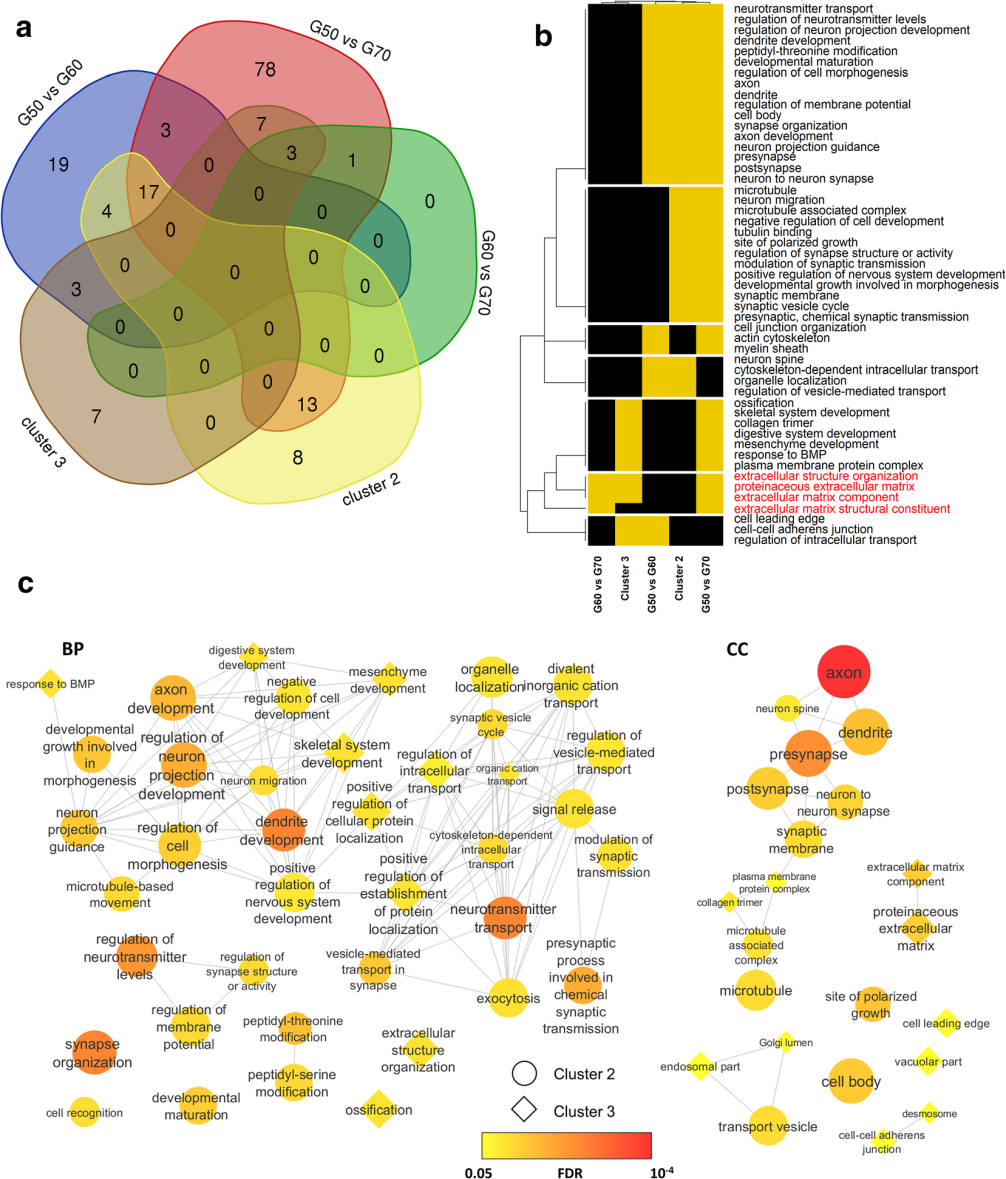
Enrichment analysis of GOs of differentially expressed genes (DEGs). **a** Venn diagram of GOs overrepresented in each analysis. **b** Heatmap of overlapping GOs in the analysis, rows represent GOs and columns represent specific analysis. Yellow color indicates that the GO is present in the specific analysis and black indicates the absence. **c** Similarity GO analysis. Each node or diamond represents a GO in Cluster 2 or 3, respectively. Sizes correspond to the number of genes observed in the category and FDR is scaled by color yellow to red according to the range of 0.05 to 10^−4^. Edges represent the similarity between the GOs

Biological enrichment analysis was performed for Cluster 3, which presented the highest expression from 50’s to 60’s (Table S5) and Cluster 2, with highest expression represented in samples at 60 years and after 75 years (Table S6). Cluster 1, with highest expression presented in samples aged ranging 60 to 75 years, showed no enrichment value. No overlap was found between GOs in Cluster 2 and Cluster 3 (Fig. 3a).

The overall biological functions in the clusters and DEG comparisons are shown in Fig. 3b. We can observe that genes related to extracellular matrix (GO categories highlighted in red) were highly expressed in G50 to G60 and differentially expressed both in G50xG70 and G60xG70, suggesting that this category probably is downregulated during aging. The same trend was observed for functions associated with membrane modifications, protein trafficking, and cell adhesion. On the contrary, functions more specifically associated with synapse modifications are upregulated with aging. Similarity analysis of GOs using NAviGO [45] with Resnik’s similarity showed differences more evident in Clusters 2 and 3 (Fig. 3c).

### Co-expression analysis

To find differences in the gene network, a preservation analysis using WGCNA was next applied. First, gene modules were set using G50 as the reference group and searched whether the topological properties from each module were preserved in the G60 group. For this analysis, only genes that were present in at least 50% of the samples from each group were selected, resulting in 12,807 genes. The modules were built using a *β* value of 38 and there were 25 gene modules (Table S7); of these, 20 were conserved and the least preserved gene modules were light cyan (*n* = 423 genes) and dark red (*n* = 354 genes) (Figure S7). Fourteen genes from light cyan were also identified as DEGs (*SYNC*, *CPT1A*, *PVRL1*, *DPH6*, *AP3B2*, *WHAMM*, *GPR61*, *LONP1*, *SCN2A*, *FBXO44*, *CAMK2B*, *STEAP4*, *FAM129B*, *MCU*), similarly for 30 in the dark red gene module (*RP11-420K14.1*, *TIMM8B*, *TBC1D15*, *NOS1*, *C14orf132*, *LINGO1*, *GNAO1*, *RNF157*, *RBFOX3*, *COLEC12*, *APC2*, *TUBB4A*, *APLP1*, *HSD17B14*, *MLC1*, *HDAC11*, *HEG1*, *PPP2R2C*, *ADTRP*, *IP6K3*, *PACSIN1*, *NRCAM*, *PTPRZ1*, *SNTG1*, *PTPRD*, *SHC3*, *NRG3*, *SCD*, *ADM*, *ARNTL*). The same approach could not be applied to G70 gene expression because it did not reach the threshold for scale-free topology. We next searched for hub genes, to select the most connected genes. First, the hub genes were compared for each sample group, to check whether the topological metrics of each least preserved module were different, between sample groups. As expected, hub genes from both modules were different between sample groups. In G50, 28 hub genes were identified in light cyan and 21 hub genes in dark red (Table 2); in the G60, 4 were identified in the light cyan and 2 in the dark red. Interestingly, 4 genes were identified both as DEGs and as hub genes in the least preserved modules (*LONP1*, *FBXO44*, *FAM129B*, *NOS1*). There was no GO enrichment for the modules least preserved in group comparison. However, the most preserved between modules midnight blue (*n* = 429 genes with 35 genes identified as DEGs) and brown (*n* = 910 genes with 88 DEGs) were related to metabolic pathways and regulatory mechanism, respectively (Table S8). The KEGG pathway analysis showed enrichment only for genes in midnight blue modules, and as expected, the metabolic pathway was overrepresented; however, genes related to degenerative diseases were also identified, such as Huntington’s, Parkinson’s, and Alzheimer’s disease (Table S9). Four genes inside these degenerative diseases also showed differential expression among ages: *CTCL* (G50 vs G60), *CALM1* (G50 vs G70), *GPR183*, and *GRIN2A* (G60 vs G70).

**Table 2:** Hub genes in each least preserved module when comparing G50 with G60

Analyses	Modules	Hubs (#genes)	
G50 vs G60	Light cyan (423genes)	G50 (28)	VN1R83P, POLE4, ZNF416, RP11-209D14.4, TIRAP, NOS3, RPUSD4, RP11-687E1.2, LINC01088, KLHL32, MTND5P26, RBM17, PCDH1, TIGD5, HERPUD2, TUBGCP5, RP11-283I3.6, C1orf131, MAGOHB, C12orf10, AC007091.1, RP11-380B4.3, LINC01023, TARSL2, *FAM129B*, MED10, CTB-55O6.8, SAE1
		G60 (4)	FBXO38, *FBXO44*, *LONP1*, RP11-278A23.1
	Dark red (354 genes)	G50 (21)	*NOS1*, TBC1D15, DHX38, ACOT2, MIR548V, DOK6, PCSK1N, CTSC, ABHD8, PRPF4, EIF3G, ZNF682, RP11-146F11.1, STUB1, CNTNAP2, RASAL2-AS1, CDK6, EXTL3, ZNF43, FAM217B, BMS1P7
		G60 (2)	EEF1D, EIF3G

Italics indicate genes differentially expressed

## Discussion

Using RNA high-throughput sequencing technology, this study characterizes the healthy adult human CP transcriptome from brain samples of individuals ranging from 50 years of age into the 80’s. The major finding pertains to the observation that the main changes seem to occur in the transition from the 50’s to the 60’s, stabilizing thereafter. It suggests that changes in the CP transcriptome occur early in the aging process and precede the time more commonly associated with brain related disabilities, such as memory and cognitive impairment. While the number of genes whose expression is altered when comparing G50 with G60 and G50 with G70 is identical, they belong to different pathways. The top 5 pathways altered between G50 and G60 are related with membrane and barrier function, being the most downregulated gene that encodes for the gap junction protein beta 2 (*GJB2*). However, when analyzing the comparison with G70, the altered pathways are related with development of axon, regulation of neuron projection, and regulation of the extracellular matrix. The latter is the only that remains altered from G60 to G70.

The age of 50’s seems to be relevant in the CP aging process. Of interest, normal aging is accompanied by a significant decrease in microvessel density from ages 55 to 85 years, and extensive arteriole tortuosity is observed after the 50’s [46]. Of relevance, cerebral blood flow is inhibited by tortuous arterioles and by the deposition of excessive collagen in the veins and venules [46], which may be related to alterations in the expression of collagen genes we observed here when comparing the G50 vs G70 and G60 vs G70. Additional studies in the previous decades will further contribute to understand whether the observed changes started even earlier.

Of notice, the data of this study pertains with the transcriptome of the entire CP, which means that it includes not only the CP epithelial cells but also the stromal space cells (endothelial cells, fibroblast, and immune cells). Altered collagen function may therefore contribute to the well-known morphological alterations described in the aged human CP that include stromal thickening, presence of hyaline bodies, calcifications, psammoma bodies, and also thickening of infiltrating arteries [9, 12]. The present study suggests that these alterations seem to occur at the molecular level as early as the age of 50’s.

Transporting substances in and out of the brain are among the best well-characterized functions of the CP. This study shows that several transport-related pathways are altered from G50 to G60. Among these are cytoskeleton-dependent intracellular transport, neurotransmitter transporter, the regulation of vesicle-mediated transport, vesicle-mediated transport to the plasma membrane, and the regulation of intracellular transport. Of interest, membrane properties may start to be compromised at this age, given the changes observed in pathways related with plasma membrane organization in the transition from G50 to G60.

It is remarkable that the changes in the CP transcriptome do not seem major, since very few genes present fold changes higher than 2. This suggests that the CP function remains considerably constant during aging, at least in healthy individuals. In accordance, when gene networks are analyzed, comparisons made between the G50 and G60 showed that most gene network modules are conserved. This highlights that alterations in midlife seem to be conserved during aging. As such, understanding modifications occurring in this period of healthy aging and their relation with later onset pathology may provide cues for targets of intervention to prevent or limit pathological aging [47, 48]. The comparisons with G70 did not reach the threshold for scale-free topology. In this network analysis, the dynamic changes during healthy aging can be represented by the following genes (differentially expressed and belonging to non-conserved networks): ion peptidase 1, mitochondrial (*LONP1*), F-box protein 44 (*FBXO44*), family with sequence similarity 129 member B (*FAM129B*), and nitric oxide synthase 1 (*NOS1*). Among these, *LONP* encodes for a mitochondrial matrix protein that mediates the selective degradation of misfolded, unassembled, or oxidatively damaged polypeptides in the mitochondrial matrix [49] and that may also have a chaperone function [50]. Reduction in mitochondria activity was observed during mouse aging [51] and the CP is enriched with mitochondria, which is required for its high secretory activity [52]. Alterations in the expression of mitochondrial proteins are likely to impact on the energy production necessary for the CSF production, described to decrease during aging. Interestingly, the *FBXO44* encodes a member of the F-box protein family that constitute one of the four subunits of the ubiquitin protein ligase complex SCFs (SKP1-cullin-F-box) that participates in phosphorylation-dependent ubiquitination. Finally, the *NOS1* gene encodes for an enzyme that synthesizes nitric oxide, which has a role in neurotransmission and may trigger oxidative damage [53].

Understanding the physiological changes that occur in aging may contribute to unravel pathways more likely to relate with diseases of high prevalence in the elderly. Among these is Alzheimer’s disease. In accordance, recent reports in the CSF proteome and in CP tissue revealed alterations in extracellular matrix pathways and oxidative stress metabolism [54, 55].

In the present study, we used clinical criteria to classify healthy controls, despite the presence of some degree of neuropathological alterations. The specialists in the field have divergent opinions on how to classify the low-burden AD neuropathologic changes seen in the great majority of cognitively healthy individuals older than 50 [56–58]. Some classify it as normal aging, whereas others consider these changes as early disease stages. This will certainly remain a matter of debate in the literature.

Altogether, this study adds to the literature that the molecular alterations that occur at the aging processes start as early as in the 50’s, with changes that are subtle but higher between the 50’s and the 60’s than thereafter. Those molecular alterations are initially related to the CP membrane function including transport and protein folding, and then progress to encompass alterations in genes that may compromise the extracellular/matrix space.

## Supplementary Information

ESM 1 (DOCX 8463 kb)

ESM 2 (XLSX 10 kb)

ESM 3 (XLSX 217 kb)

ESM 4 (XLS 25 kb)

ESM 5 (XLSX 38 kb)

ESM 6 (XLSX 13 kb)

ESM 7 (XLSX 17 kb)

ESM 8 (XLSX 607 kb)

ESM 9 (XLSX 16 kb)

ESM 10 (XLS 29 kb)

## Data Availability

The raw data of RNA-seq in this study is available in SRA repository under the number accession PRJNA515530 and study SRP179998.
