# Supplementary material for: Are the 50’s, the transition decade, in choroid plexus aging?
Source: GeroScience. 2021 Feb 12;43(1):225–37. doi: 10.1007/s11357-021-00329-x (PMC8050122; doi:10.1007/s11357-021-00329-x)
Supplement: Supplementary file 1 — (DOCX 8463 kb) [file 11357_2021_329_MOESM1_ESM.docx]

**Supplementary file:**

Sequenced reads were mapped using Hisat2 [1,2] and quality mapping were verified using RSeQC [3]. One of the parameters that were checked was coverage along the gene to identify if there were any bias in gene coverage. As expected, no bias was observed along the gene coverage, which means that most part of genes measured are spread along the gene body (Figure S1). In order to check if all samples were comparable according to gene count measure, Pearson's correlation was calculated, and all samples presented r ≥ 0.8 (Figure S2). In order to inspect if samples could group according to one of the variables studied, we clustered samples according to gene expression and plotted the variables according to sample clustering. No bias according to sample is observed in the group (Figure S3). Regarding gene expression, about 54% of genes (32,672) were detected in at least one sample, however 30% of genes (18,122) were detected in at least 10 samples with only 21% (12,528) measured in all samples in the study (Figure S4). After applying low expression filter (CPM ≥ 0.3), only 15,319 genes (25.32%) remained.


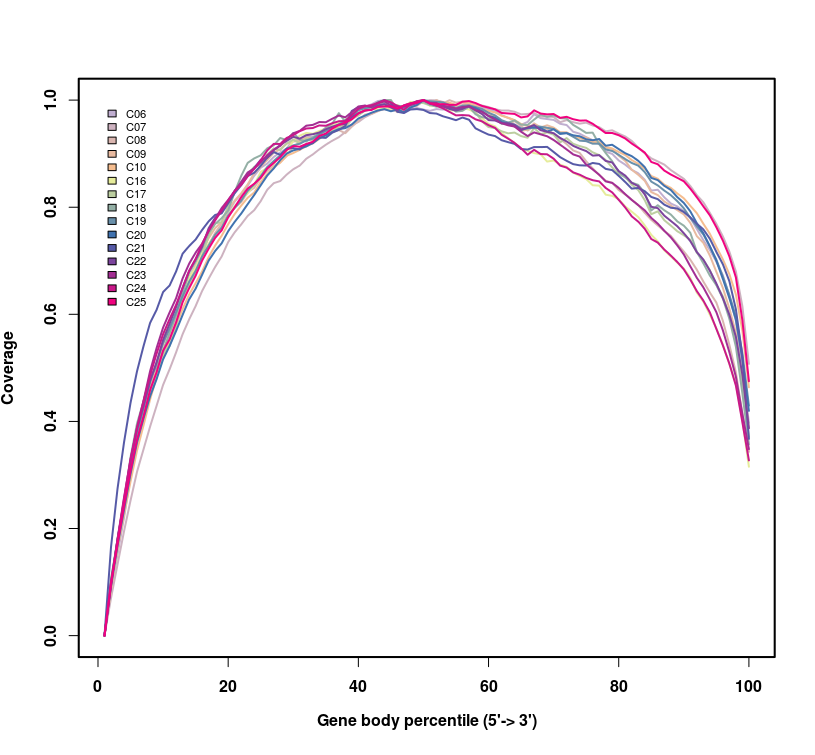


Figure S1: Gene body coverage. The x axis represents the gene body percentile split into 1 to 100 and y axis the coverage in each percentile range.


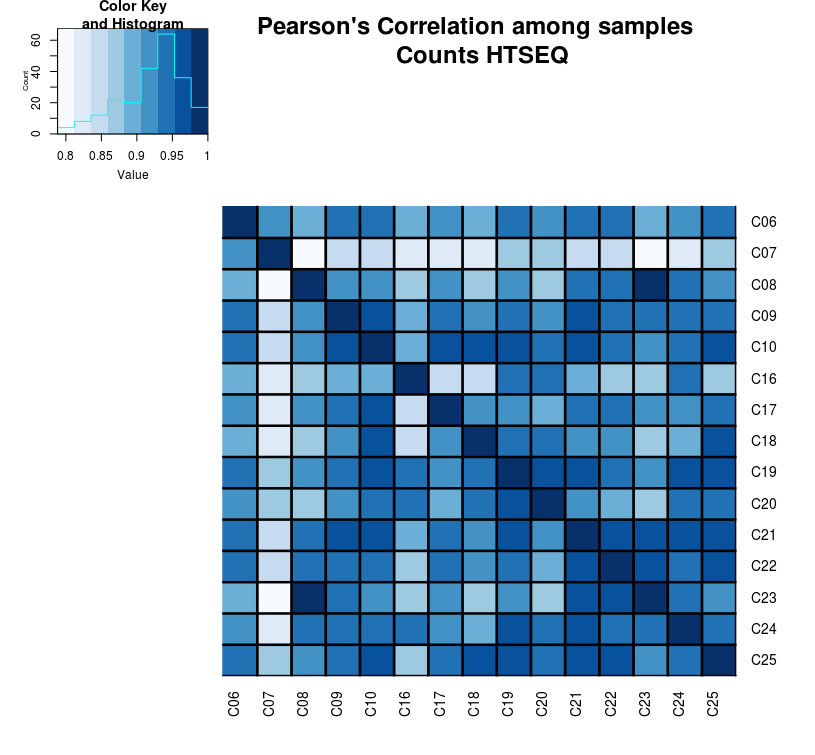


Figure S2: Heatmap of Pearson's correlation coefficient among samples. Each line and each column represents a sample, and color are scaled according to Pearson’s Correlation Coefficient (PCC).


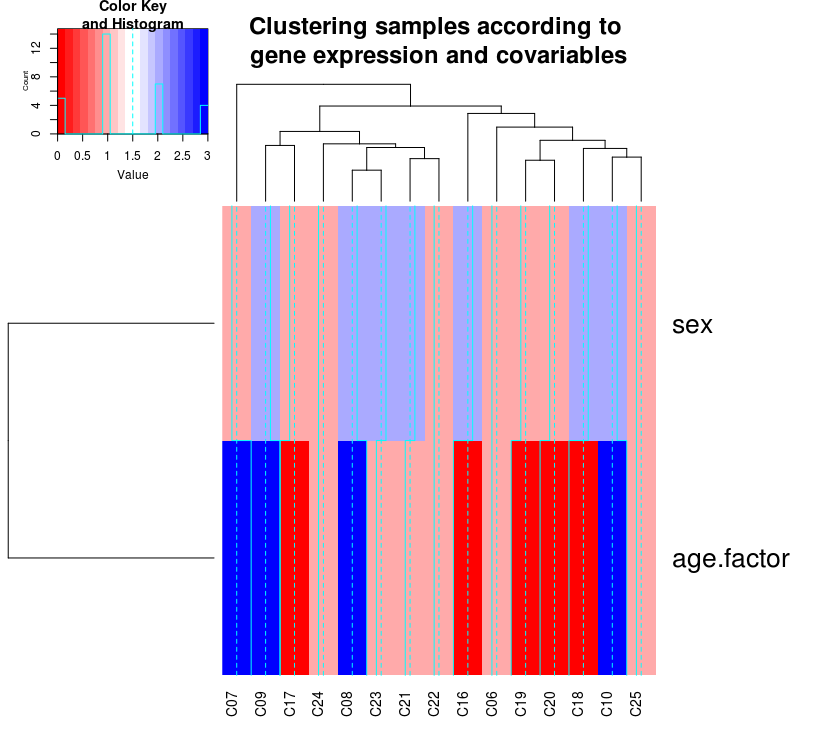


Figure S3: Sample clustering according to gene expression using Euclidean distance. Covariables of each sample is represented using the same clustering pattern gene expression. The covariables: sex (1 – Male/pink and 2 – Female/blue), age.factor (0 - < 60 years/red, 1 - ≤ 70 years/pink, 2 - ≤ 80/light blue and 3 - > 90/blue).


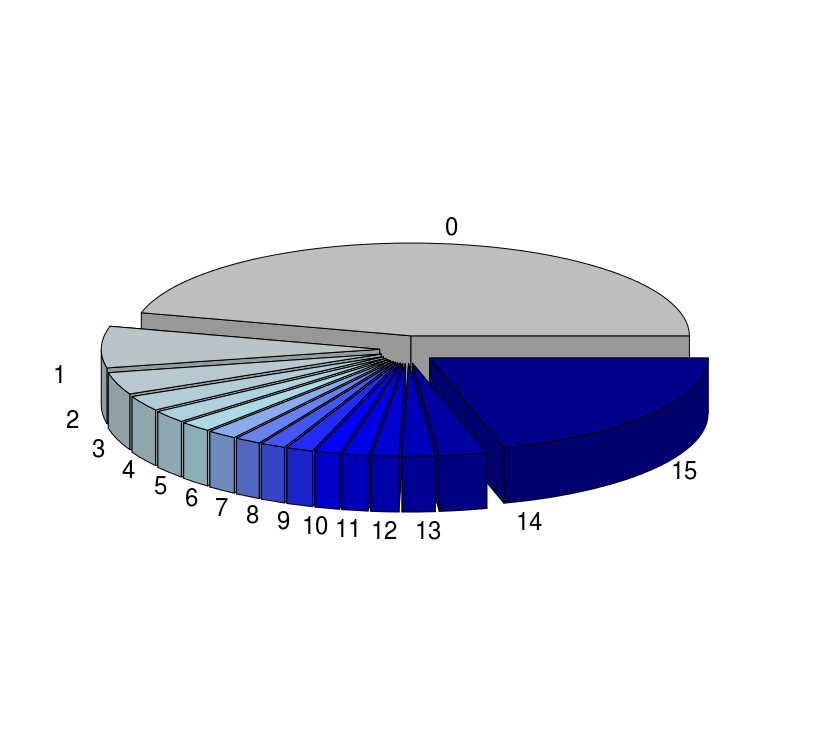


Figure S4: Pie chart of genes expressed according to the number of samples. 0 indicates that the gene was not detected in any sample studied 46% (27,832) and 15 indicates that the gene was measured in all samples studied, resulting in 21% of total (12,528).

The total within sum of squares shows the sum of variances in all groups identified. Variance inside each group decreases while k (number of groups) increases, because all the points inside the group are closer to the centroid. However, at a certain point of k the decrease of variance reaches a plateau (Fig S5, red line), which means the best number of groups to split the data.


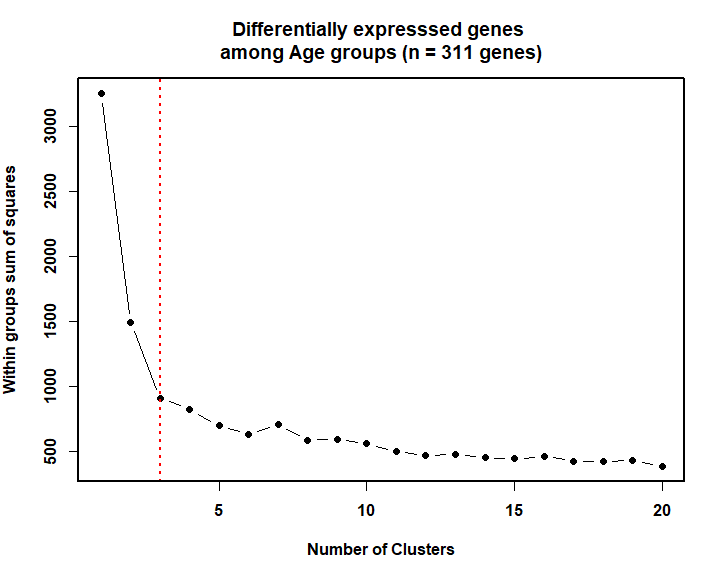


Figure S5: Variance within groups by number of clusters. The y-axis shows the within group variance and x-axis the number of groups (clusters). The red line indicates the best number of groups according to variance

**References:**

1. Sirén J, Välimäki N, Mäkinen V. HISAT2 - Fast and sensitive alignment against general human population. IEEE/ACM Trans Comput Biol Bioinforma. 2014;11:375–88.

2. Pertea M, Pertea GM, Antonescu CM, Chang TC, Mendell JT, Salzberg SL. StringTie enables improved reconstruction of a transcriptome from RNA-seq reads. Nat Biotechnol. 2015;33:290–5.

3. Benjamini Y, Speed TP. RSeQC: Quality Control of RNA-seq experiments. Bioinformatics. 2012;40:e72.
